# Venom peptides in association with standard drugs: a novel strategy for combating antibiotic resistance - an overview

**DOI:** 10.1590/1678-9199-JVATITD-2020-0001

**Published:** 2020-08-10

**Authors:** Ashish K. Lamiyan, Ramkesh Dalal, Neelima R. Kumar

**Affiliations:** 1 Department of Zoology, Panjab University, Chandigarh, India.

**Keywords:** Antibiotic resistance, Antimicrobials, Venom

## Abstract

Development of antibiotic resistance that leads to resurgence of bacterial infections poses a threat to disease-free existence for humankind and is a challenge for the welfare of the society at large. Despite research efforts directed towards treatment of pathogens, antibiotics within new improved classes have not emerged for years, a fact largely attributable to the pharmacological necessities compelling drug development. Recent reversion to the use of natural products alone or in combination with standard drugs has opened up new vistas for alternative therapeutics. The success of this strategy is evident in the sudden interest in plant extracts as additives/synergists for treatment of maladies caused by drug-resistant bacterial strains. Animal venoms have long fascinated scientists as sources of pharmacologically active components that can be exploited for the treatment of specific ailments and should be promoted further to clinical trials. In the present review, we outline the scope and possible methods for the applications of animal venoms in combination with commercial antibiotics to offer a better treatment approach against antibiotic-resistant infections.

## Background

Antibiotics are the chemical entities that kill bacteria or slow down their growth. However, these one-time wonder medicines of the antibiotic era were not without serious side effects. It has now been established that long term use and overuse of antibiotics have given rise to a serious complication known as *antimicrobial resistance* [1]. When penicillin, a naturally occurring antibiotic, was discovered in 1929 by Fleming, microbial-derived antibiotics brought a complete revolution in antimicrobial therapeutics and became the main line of defense against infectious diseases [1, 2]. 

Despite recent advances in the field of modern medicine, bacteria still impose great risks to human health. Moreover, resistance emerged against many classes of commonly used antibiotics giving rise to multidrug resistance (MDR) [2]. The unresolved status of resistance mechanisms has become such a matter of concern that the World Health Organization (WHO) considers it urgent to require the development of alternative therapeutics due to drug resistance. 

Bacteria have been successful in developing resistance by means of different mechanisms including modification in their genes, an option for survival adopted by both pathogenic and non-pathogenic microorganisms [3]. The high level of regular use and overuse of commercial antibiotics complicates the situation and hampers the effectiveness of drugs developed by the pharmaceutical industry [1]. In the existing scenario, it is required to test the presently established line of drugs and work diligently to fill the gap between new drug discovery and the rising need for alternatives to combat antimicrobial resistance [4]. 

In the light of the fact that there was fast development of resistance against single-agent compounds (monotherapy) that target essential enzymes only, it was deemed urgent to develop antibiotics that act upon multiple targets. Then, two new classes of antibiotic agents entered the market in the last three decades, the oxazolidinones and lipopeptides [5]. The development of the multitargeted antibiotics was due to the rise of resistance against the earlier ones such as sulfonamide drugs introduced in 1930s [6]. 

The antibiotic resistance issue has propelled the examination of new alternative medications for bacterial infection control with synergistic effects [7, 8]. Since ancient times humankind has benefitted from natural products for antibiotic therapies [9]. With the rapid increase in bacterial resistance against antibiotics, scientific efforts have been redirected towards a search for alternatives from nature that are potent but also less toxic. The present review focuses on antibiotic resistance, antimicrobial activity of animal venoms and strategies for the development of new first-line antibiotic therapies. In this context, animal venoms can be viewed, particularly in synergistic combinations, as a better option for rapidly developing a new line of antibiotics for combating pathogens resistant to conventional antibiotic therapeutics.

## Methods

### Search strategy

A systematic review was carried out following the rules and guidelines of PRISMA (Preferred Reporting Items for Systematic Reviews and Meta-Analysis) [10]. PubMed and Google Scholar were the electronic databases exclusively searched for articles published on antibiotic resistance and antimicrobials from venoms. No limit on publication dates was set. The literature search was initiated on March 1, 2019 with an update on September 30, 2019. The reference list of relevant articles was checked for additional titles for inclusion in the review. The literature was examined utilizing a search string containing combinations of terms including: “burden”, “antibiotic”, “antimicrobial”, “multi-drug”, “microbial-drug”, “resistance”, “gram-positive”, “gram-negative”, “venom”, “combination”, “additive” and “synergistic”.

### Study selection

The studies were selected by the cooperation of two reviewers (AKL and RD) through the software Endnote (version X9, Clarivate Analytics, 2017) and verified by a third reviewer (NRK) ensuring the specificity and quality of the process. The literature was chosen on the basis of the following criteria: full-text accessible articles in which experimental studies were carried out; in an examination two antimicrobial peptides (AMPs), denominated La47 and Css54, from spider (*Lachesana* sp*.*) and scorpion (*Centruroides suffusus*), blends of La47 with the antibiotic agents like chloramphenicol, streptomycin and kanamycin, showed the best antimicrobial results. Likewise, the other novel peptide Css54 - when assessed with respect to antibiotic agents utilized for tuberculosis treatment, isoniazid, rifampicin, pyrazinamide and ethambutol - showed the best results with rifampicin [11]. Another study reported an improvement against the bacterial growth (*S. aureus* and *P. aeruginosa*) when macropin was given in combination with commercial antibiotic at a lower dose as compared to the peptide or antibiotics used alone [12]. Similarly, an additive effect was observed against *P. aeruginosa* strains treated with combinations of macropin and various antibiotics. The combination of oxacillin with macropin (for *S. aureus*) and piperacillin with macropin (for *P. aeruginosa*) increased the bacteriostasis rate very rapidly indicating a strong inhibitory potential [13]. 

### Data extraction

The literature for inclusion in the review was assessed by two independent reviewers (AKL and RD), who chose the studies based on the parameters required. The inclusion of articles was restricted to a very limited set of selected pathogens on the basis of drug resistance. The discussed sections included the action mechanism of the venom peptides with respect to membrane permeabilization and the lipopolysaccharide-binding phenomenon. The possibility of inconsistency was discussed by the contributing authors until reaching a final conclusion. The data extracted from the included articles contained the following: the author’s name and the year of publication; the type of disease; study design; random methods; treating method of antimicrobial involvement; treatment method and primary outcomes.

## Results

**Figure 1.**
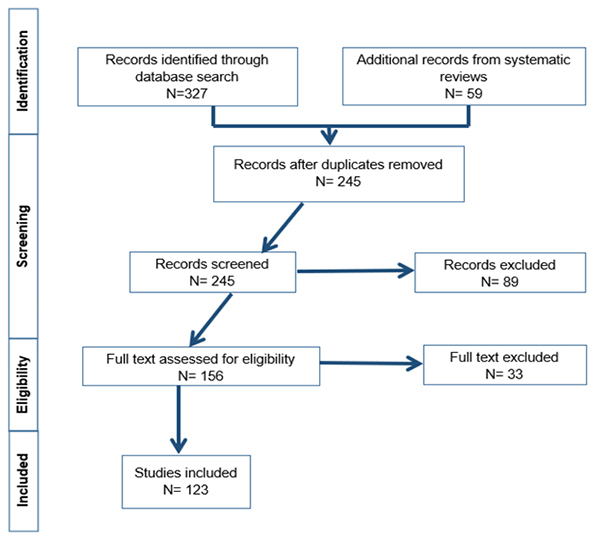
PRISMA flowchart showing the study design process.

Based on the selection process, out of the 327 total titles and abstracts retrieved over the specified search period, 123 studies were included in the final review (Fig. 1). Many of the studies included summaries of articles presented, experimental studies or review articles. The duplicate records and unrelated literature was excluded from the selection process. The PRISMA flowchart of the study plan is shown in Figure 1. The recorded data included author name, year of publication, country, type, sample size, bacterial species and drugs. The above details were extracted separately by two researchers (AKL and RD).

### Mechanism of action for venoms

Venoms are complex chemical entities that comprise several components containing biologically active molecules. Snakes, scorpions, bees, wasps, centipedes and frogs are some animals that use venom for defending themselves or for capturing prey. These venoms or toxins, however, vary in composition and action mechanism from species to species. Certain peptides present in venom have been reported as being capable of causing damage to cellular membrane of microbes through electrostatic attraction forces [14, 15]. The bacterial cell surfaces are generally negatively charged, a property solely responsible for the selective binding of AMPs with the bacterial cell membranes due to the presence of AMP-positive AA residues [16]. The mechanism of action of antimicrobial peptides (AMPs) derived from different animal venoms presents different working cascades. The difference in working mechanisms is due to factors including physiochemical properties of the peptides and composition of the lipids in membrane of the microbial pathogens [17]. There are many mechanisms that explain how the pore formation processes cause expeditious disintegration of the bilayer structures present in cell membrane of a microbial pathogen [18]. Previous studies have revealed the presence of complex hydrophobic proteins and peptides (myotoxic phospholipases, neurotoxins, latarcins) as secondary structures in forms such as a-helixes or b-sheets [19]. It is highly essential to understand their key role in the crucial phenomenon of pore formation and membrane-degrading effects.

### Membrane permeabilization

Several studies have been carried out on venom proteins and peptides isolated from snakes (reptiles) for exploring their antimicrobial activity. A study was carried out on CaTx-II, a type of phospholipase isolated from venom of the snake *Crotalus adamanteus.* It showed inhibition against *Staphylococcus aureus* at the concentration of 7.8 mg/ml, and against *Bacillus pseudomallei* and *Enterobacter aerogenes* at the concentration of 15.6 mg/ml. It was further reported that CaTx-II induced pore formation and membrane-damaging effects on the bacterial cell wall but caused no cytotoxicity to fibroblast cells isolated from skin and lung tissues [20]. Furthermore, another peptide, Smp24, derived from the venom of the scorpion *Maurus palmatus*, which is usually 24 amino acids in length and carrying a triple positive charge, showed lethal potential against microbes. Smp24 also induced formation of pores with continuous increase in concentration and caused destruction of lipid bilayers, clearly indicating a phospholipid-dependent phenomenon [21]. In addition, conotoxins, a type of amino acids rich in glycine and cysteine, have also been reported, thereby suggesting that the flexibility of structure in relation to aromatic residues and membrane interaction by hydrophobic attraction was due to the presence of these peptides. AMPs bearing positive charge/ net hydrophobic charges have flexible chain structures and are crucial for the development of inhibitory potential against pathogens [22].

Snake venom enzymes like PLA2-derived peptides resulted in permeabilization of the bacterial cell membrane, demonstrating that the peptides possessed bactericidal effects [23]. Simultaneously, these peptides blocked the effect of bacteria on macrophages and other target cells of the infected host by combining with bacterial lipopolysaccharides. Another study for understanding the mechanisms underlying the formation of pores by the venoms was performed on the sea anemone *Stichodactyla healiantus* with stychoysin I peptide (St-I) where the rate of permeabilization increased with the increment of sphingomyelin (SM) into phosphatidylcholine (PC), which was attributed to toxin binding [24].

### Binding to bacterial lipopolysaccharides (LPS)

Lipopolysaccharides (LPS) form an important constituent of the external membrane of Gram-negative bacteria and are functionally protective in nature. Due to this fact, the interaction of LPS with LPS-binding molecules attracts great attention in the development of antibiotics. An example of such an interaction is demonstrated by antimicrobial peptides (AMPs), which have a very high affinity for LPS in the bacterial membrane. The susceptibility of bacteria to the AMPs is confirmed by the biophysical properties of AMPs and their mode of interaction with LPS of the membrane [25].

In recent studies, peptides derived from phospholipases (PLA2) from snake venom also revealed interaction with a specific lipid component of various Gram-negative bacteria leading to death of the pathogen [26]. In another recent work, macropin was isolated from bee venom. Both Gram-positive and -negative bacteria exhibited inhibition by this antimicrobial component of the venom. Macropin was found to bind with the peptidoglycans and lipopolysaccharides and killed the bacteria by disruption in their membranes suggesting that it had antimicrobial potential and could be used as a bactericidal agent for infectious drug-resistant bacteria [27]. Furthermore, the fractional inhibitory concentration index obtained in the experimental observations indicated that the component had additive and partially synergistic effects with conventional antibiotics against various drug-resistant bacteria [13]. 

The phospholipases A2 (PLA2), i.e., myotoxins II (Lys49) and III (Asp49), isoforms isolated from the venom of *Bothrops asper* inflammatory fluids, revealed bactericidal potential. The study shown a higher binding affinity of the PLA2 isoforms to the isolated lipopolysaccharide (LPS) of susceptible bacteria [28, 29]. Mastoparans (MPs) are one of the other antimicrobial peptides that are isolated from the wasp venom and show cationic and amphiphilic properties [30, 31]. These balance different cell functions, including incitement of GTP-restricting protein, phospholipase C and can tie to a phospholipid bilayer [30]. Mastoparan-1 (MP-1), another tetradecapeptide poison separated from hornet venom, was produced synthetically for an examination where Escherichia coli (*E. coli* 25922) and LPS were utilized for inducing sepsis in a murine model. It was discovered that MP-1 treatment at a rate of 3 mg/kg produced a defensive impact on mice from the general disease condition induced by the microscopic organisms and LPS challenges. MP-1 has antibacterial capacities against gram-negative and gram-positive bacteria, which may be due to the destructive action of the AMPs toward the bacterial membrane structure. In addition, respiratory burst inhibition was prominent during the treatment of murine peritoneal macrophages with MP-1 specifically. This effect could be attributed to the inhibition of NADPH oxidase in the film. Moreover, MP-1 additionally reduced the expression of TLR4, TNF-alpha and IL-6 mRNA and the formation of cytokines in LPS-administered murine peritoneal macrophages, thus demonstrating protective potential against deadly microorganisms revealing the bactericidal activity of AMPs, which limited the reactions of macrophages to the two microscopic organisms and LPS [32].

### Antibacterial peptides of venoms

The ability of venom proteins to bind to the lipid component of the cell membrane of several bacteria led to a series of extensive searches to isolate active proteins from animal poisons, which could be used alone or as additives and synergists with standard drugs in order to combat resistant bacteria. Some of the related studies that investigated the antibacterial peptides present in venom are displayed in Table 1.

**Table 1.** Antibacterial peptides of venoms.

	Microbe	Resistant to	Effective venom peptides	Source	Reference
1	*Alacaligenes faecalis*	Aminoglycosides, chloramphenicol and tetracyclines	Ponericin (G1, G3, W1, W3-desK, W4)	*Pachycondyla geoldii*	[33, 34]
2	*Acinetobacter baumannii.*	Quinolone, beta-lactam/beta-lactamase inhibitor, cephalosporin and carbapenem	Vejovine Crotalicidin Batroxicidin Mastoparan Lycosin II OH-CATH30	*Vaejovis mexicanus,* *Crotalus durissus terrificus,* *Bothrops atrox,* *Vespa basalis,* *Vespa lewisii,* *Lycosa singoriensis,* *Ophiophagus hannah*	[35-40]
3	*Bacillus cereus*	Beta-lactam antibiotics such as ampicillin, oxacillin, penicillin, amoxicillin, and cefepime	Ponericin (G4, G6, L2, W1, W3-desK, W4, W5, W6) Melectin Polybia (MP-II, MP-III) Defensin-NV Wa-PLA2	*Pachycondyla geoldii,* *Melecta albifrons,* *Polybia paulista,* *Nasonia vitripennis,* *Walterinnesia aegyptia*	[12, 34, 41-44]
4	*Bacillus subtilis*	Chloramphenicol, tetracycline, rifampicin and streptomycin	Ponericin (G1, G3, G4, G6, L2, W1, W3-desK, W4, W5, W6, Q42) Lasioglossin (LL-I, LL-II, LL-III) Lasiocepsin Halictine (1, 2) Macropin Panurgine I (PNG-I, K, R) Codesane Scorpine Heteroscorpine-I Bactridine (1, 2) 5Opistoporin 1 Pandinin (1, 2) Parabutoporin Mucroporin Imcroporin Ctriporin Lycocitin (I, II) Latarcin (1, 2a, 3a, 3b, 4a, 4b, 5, 6a, 7) Cyto-insectotoxin 1a Latartoxin 1a Mastoparan Mastoparan (B, PDD-A, PDD-B, MP, PMM) Agelaia-MP Protonectin Polybia-CP Anoplin Eumenitin Decoralin Crabrolin Dominulin (A, B) Wa-PLA2	*Pachycondyla geoldii,* *Ectatomma quadridens,* *Lasioglossum laticeps, Lasioglossum laticeps, Halictus sexcinctus,* *Macropis fulvipes,* *Panurgus calcaratus,* *Colletes daviesanus,* *Pandinus imperator,* *Heterometrus* *laoticus,* *Tityus discrepans,* *Opistophtalmus carinatus,* *Pandinus imperator,* *Opistophtalmus carinatus,* *Lychas* *mucronatus,* *Isometrus maculates,* *Chaerilus tricostatus,* *Lycosa singoriensis,* *Lachesana tarabaevi,* *Vespa basalis,* *Vespa lewisii,* *Polistes dorsalis dorsalis,* *Mischocytarus phthisicus,* *Polistes major major* *Agelaia pallipes pallipes,* *Polybia paulista,* *Anoplius samarienis, Oreumenes* *decorates,* *Eumenes rubronotatus,* *Vespa crabro,* *Polistes dominulus,* *Walterinnesia aegyptia*	[27, 34, 37, 38, 41, 44-70]
5	*Bacillus thuringiensis*	Amoxicillin and ampicillin	Lycotoxin (I, II) Anoplin Eumenitin Decoralin	*Lycosa carolinensis,* *Anoplius samariensis,* *Eumenes rubronotatus,* *Oreumenes decorates*	[67-69, 71, 72]
6	*Citrobacter freundii*	Penicillins, cephalosporins, ciprofloxacin, levofloxacin, aminoglycosides, phenicols, sulfonamides, tetracyclines and nitrofuran	Pilosulin 1 Melittin	*Myrmecia pilosula,* *Apis mellifera*	[73-80]
7	*Enterobacter cloacae*	Beta-lactamases or carbapenemases ampicillin, amoxicillin-clavulanic acid, cephalothin, and cefoxitin	Ponericin G1, G3, L2, W1, W3-desK, W4, W5 Hadrurin Vejovine Heterin (1, 2) Pantinin (1, 2, 3) Spiniferin Mastoparan (VT1, VT2, VT3, VT4, VT5, VT6) Anoplin Eumenitin	*Pachycondyla geoldii,* *Hadrurus aztecus,* *Vaejovis mexicanus,* *Heterometrus spinifer,* *Pandinus imperator,* *Vespa basalis,* *Vespula lewisii,* *Anoplius samariensis, Eumenes rubronotatus*	[34, 56, 65, 67, 68, 81-84]
8	*Escherchia coli*	Ampicillin, amoxicillin, tetracycline, co-trimoxazole, streptomycin, ciprofloxacin, ofloxacin, cefotaxime, and gentamicin, chloramphenicol	Ponericin (G1, G3, L2, W1, W3-desK, W4, W5, Q42, Q49, Q50) Pilosulin 1 Melittin Melectin Lasioglossin (LL-I, LL-II, LL-III) Lasiocepsin	*Pachycondyla geoldii,* *Myrmecia pilosula,* *Apis mellifera,* *Melecta albifrons,* *Lasioglosssum laticeps*	[22, 23, 28, 29, 34, 36, 37, 38, 41-44, 46-52, 55-59, 62-70, 72, 73, 75-80, 82, 83-96]
			Halictine (1, 2) Macropin Panurgine I PNG-I, K, R) Codesane Scolopin (1, 2) Conolysin-Mt ω-conotoxin MVIIA Opiscorpine 1 Opistoporin 1 Parabutoporin Pandinin (1, 2) Hadrurin Vejovine Heterin (1, 2) Meucin (13, 18) Mucroporin Imcroporin Ctriporin Pantinin (1, 2, 3) Spiniferin Stigmurin Mauriporin Crotamin Crotalicidin Batroxicidin Lycotoxin (I, II) Lycocitin (I, II) Latarcin (2a, 3a, 3b, 4a, 4b, 5, 6a, 7) Cyto-insectotoxin 1a Latartoxin 1a Mastoparan Mastoparan (B, X, VT1, VT2, PDD-A, PDD-B, MP, PMM) Polybia-MP (I, II, III) Agelaia-MP Protonectin Polybia-CP Anoplin Eumenitin Decoralin Crabrolin Dominulin (A, B) Defensin-NV Myotoxin (II, III) Wa-PLA2 CTX-3 CTX-1	*Halictus sexcintus,* *Macropis flavus,* *Panurgus calcaratus,* *Colletes daviesanus,* *Scolopendra subspinipes,* *Conus mustelinus,* *Conus* spp., *Opistophthalmus carinatus,* *Pandinus imperator,* *Hadrurus aztecus,* *Vaejivis mexicanus,* *Heterometrus spinifer,* *Mesobuthus eupeus,* *Lychas mucronatus,* *Isometrus maculates,* *Chaerilus tricostatus,* *Tityus stigmurus,* *Androctonus mauritanicus,* *Crotalus durissus,* *Crotalus durissus terrificus,* *Bothrops atrox,* *Lycosa carolinensis,* *Lycosa singoriensis,* *Lachesana tarabaevi,* *Vespa basalis,* *Vespa lewisii,* *Vespa* spp., *Polybia paulista,* *Agelaia pallipes pallipes,* *Anoplius samariensis, Oreumenes decorates,* *Eumenes rubronotatus,* *Vespa crabro,* *Polistes dominulus,* *Nasonia vitripennis,* *Bothrops asper,* *Walterinnesia aegyptia,* *Naja naja atra,* *Naja naja*	
9	*Enterococcus faecalis*	Tetracycline, erythromycin, ampicillin and ciprofloxacin	Ponericin (G1, G3, L2, W1, W3-desK, W4, W5, W6) Melittin Bactridine (1, 2) Opistoporin 1 Hadrurin Pandinin (1, 2) Parabutoporin Crotalicidin Batroxicidin Mastoparan Mastoparan (B, VT1, VT2, VT3, VT6, VT7) Decoralin	*Pachycondyla geoldii,* *Apis mellifera,* *Tityus discrepans,* *Opistophtalmus carinatus,* *Hadrurus aztecus,* *Pandinus imperator,* *Opistophtalmus carinatus,* *Crotalus durissus terrificus,* *Bothrops atrox,* *Vespa basalis,* *Vespa* spp., *Oreumenes decorates*	[34, 36, 37, 54-57, 69, 75-80, 84, 97]
10	*Haemophilus influenza*	Ampicillin, cefuroxime, clarithromycin, cefaclor, amoxicillin-clavulanate, and chloramphenicol	Opistoporin 1 Parabutoporin	*Opistophtalmus carinatus*	[55, 98]
11	*Klebsiella pneumonia*	Trimethoprim/sulfamethoxazole, ceftriaxone, tobramycin, ciprofloxacin, piperacillin/tazobactam, ceftazidime, aztreonam.	Ponericin (G1, G3, G4, G6, L2, W1, W3-desK, W4, W5) Pilosulin 1 Opistoporin 1 Hadrurin Parabutoporin Vejovine Mauriporin Crotalicidin Batroxicidin Mastoparan Mastoparan (B, VT1, VT2, VT3, VT4, VT6, VT7) Decoralin	*Pachycondyla geoldii,* *Myrmecia pilosula,* *Opistophtalmus carinatus,* *Hadrurus aztecus,* *Vaejivis mexicans,* *Androctonus mauritanicus,* *Crotalus durissus terrificus,* *Bothrops atrox,* *Vespa basalis,* *Vespa* spp., *Oreumenes decorates*	[34, 36-38, 44, 55, 56, 69, 73, 82, 85, 98, 99]
12	*Pseudomonas aeruginosa*	Fosfomycin, ciprofloxacin, levofloxacin, ceftazidime, piperacillin, imipenem, piperacillin, tobramycin, gentamicin, and meropenem	Ponericin (G1, G3, G4, G6, L2, W1, W3-desK, W4, W5, W6, Q42) Pilosulin 1 Melittin Melectin Lasioglossin (LL-I, LL-II, LL-III) Halictine (1, 2) Lasiocepsin Macropin Panurgine I (PNG-I, K, R) Codesane Bactridine (1, 2) Opistoporin 1 Hadrurin Pandinin (1, 2) Parabutoporin Vejovine Mucroporin Imcroporin Ctriporin Mauriporin Crotamin Crotalicidin Batroxicidin Lycocitin II Latarcin (2a, 3a, 3b, 4a, 4b, 5, 6a, 7) Cyto-insectotoxin 1a Latartoxin 1a Mastoparan (VT1, VT2, VT3, VT4, VT6, VT7) Polybia-MP (I, II, III) Agelaia-MP Protonectin Polybia-CP Anoplin Eumenitin Defensin-NV Wa-PLA2 Lmut Tx CTX-1	*Pachycondyla geoldii,* *Myrmecia pilosula,* *Apis mellifera,* *Melecta albifrons,* *Lasioglossum laticeps,* *Halictus sexcinctus,* *Macropis fulvipes,* *Panurgus calcarutus,* *Colletes daviesanus,* *Tityus discrepans,* *Opistophthalmus carinatus,* *Hadrurus aztecus,* *Pandinus imperator,* *Vaejivis mexicans,* *Lychas mucronatus,* *Isometrus maculates,* *Chaerilus tricostatus,* *Androctonus mauritanicus,* *Crotalus durissus,* *Crotalus durissus terrificus,* *Bothrops atrox,* *Lycosa singoriensis,* *Lachesana tarabaevi,* *Vespa* spp., *Polybia paulista,* *Agelaia pallipes pallipes,* *Polybia paulista,* *Anoplius samariensis,* *Eumenes rubronotatus,* *Nasonia vitripennis,* *Walterinnesia aegyptia,* *Lachesis muta muta,* *Naja naja*	[23, 27, 34, 36, 38, 41-44, 46-51, 54-59, 61-68, 73, 75-80, 82, 84, 96, 100, 101]
13	*Pseudomonas flourescens*	Meropenem, piperacillin/tazobactam and ceftazidime	Melittin Heterin (1, 2) Spiniferin Cyto-insectotoxin 1a	*Apis mellifera,* *Heterometrus spinifer,* *Lachesana tarabaevi*	[63, 75-80, 83, 102]
14	*Proteus mirabilis*	Fluoroquinolone, cephalosporin, gentamicin, trimethoprim-sulfamethoxazole, gentamicin	Ponericin (G1, G3) Melittin Anoplin Eumenitin	*Pachycondyla geoldii,* *Apis mellifera,* *Anoplius samariensis,* *Eumenes rubronotatus*	[34, 67, 68, 75-80, 102]
15	*Psuedomonas putida*	Ciprofloxacin, norfloxacin, pefloxacin and ofloxacin, gentamycin, kanamycin, neomycin, streptomycin and netilmicin	Ponericin (G1, G3, W1, W3-desK, W4) Heterin (1, 2) Pantinin (1, 2, 3) Spiniferin	*Pachycondyla geoldii,* *Heterometrus spinifer,* *Pandinus imperator,* *Heterometrus spinifer*	[34, 83, 92, 103]
16	*Staphylococcus aureus*	Beta-lactams, glycopeptides, aminoglycosides, quinolones, oxazolinidones	Ponericin (G1, G3, G6, W1, W3-desK, W4, W5, W6) Bicarinalin Pilosulin 1 Melittin Melectin Lasioglossin (LL-I, II, III) Halictine (1, 2) Lasiocepsin Macropin Panurgine I (PNG-I, K, R) Codesane Scolopin (1, 2) Conolysin-Mt ω-conotoxin MVIIA Opistoporin 1 Pandinin (1, 2) Parabutoporin Heterin (1, 2) Mucroporin Imcroporin Ctriporin Pantinin (1, 2, 3) Spiniferin Crotamin Crotalicidin Batroxicidin Cyto-insectotoxin 1a Mastoparan Mastoparan (B, VT1, VT2, VT3, VT4, VT6, VT7) Polybia-MP (I, II, III) Agelaia-MP Protonectin Polybia-CP Anoplin Eumenitin Decoralin Crabrolin Defensin-NV Myotoxin (II, III) Wa PLA2 PnPLA2 CTX-3 Lmut Tx OH-CATH30 CTX-1	*Pachycondyla geoldii,* *Tetramorium bicarinatum,* *Myrmecia pilosula,* *Apis mellifera,* *Melecta albifrons,* *Lasioglossum laticeps,* *Halictus sexcinctus,* *Lasioglossum laticeps,* *Macropis fulvipes,* *Panurgus calcaratus,* *Colletes daviesanus,* *Scolopendra subspinipes mutilans,* *Conus mustelinus,* *Conus* spp., *Opistophthalmus carinatus,* *Pandinus imperator,* *Heterometrus spinifer,* *Lychas mucronatus,* *Isometrus maculates,* *Chaerilus tricostatus,* *Crotalus durissus,* *Crotalus durissus terrificus,* *Bothrops atrox,* *Lachesana tarabaevi,* *Vespa basalis,* *Vespa* spp., *Polybia paulista,* *Agelaia pallipes pallipes,* *Anoplius samariensis,* *Eumenes rubronotatus,* *Oreumenes decorates,* *Vespa crabro,* *Nasonia vitripennis,* *Bothrops asper,* *Walterinnesia aegyptia,* *Porthidium nasutum,* *Naja naja atra,* *Lachesis muta muta,* *Ophiophagus hannah,* *Naja naja,*	[22, 23, 27-29, 34, 36-38, 40-44, 47-51, 55, 57-59, 63, 65-70, 73, 75-80, 83, 84, 87, 88, 90, 92, 95, 96, 104, 105]
17	*Salmonella enterica*	Fluoroquinolones, cephalosporins	Ponericin (G1, G3, W1, W3-desK) Melittin Heterin (1, 2) Pantinin (1, 2, 3) Spiniferin Stigmurin Mauriporin Wa PLA2	*Pachycondyla geoldii,* *Apis mellifera,* *Heterometrus spinifer,* *Pandinus imperator,* *Tityus stigmurus,* *Androctonus mauritanicus , Walterinnesia aegyptia*	[34, 44, 75-80, 83, 85, 92, 93, 106]
18	*Staphylococcus epidermidis*	Methicillin, nafcillin, penicillin, cephalothin, cefamandole, streptomycin, and gentamicin	Ponericin (G1, G3, G6, L2, W1, W3-desK, W4, W5, W6) Pilosulin 1 Pandinin(1, 2) Ctriporin Mauriporin Mastoparan Mastoparan B Polybia-CP Eumenitin Wa PLA2	*Pachycondyla geoldii,* *Myrmecia pilosula,* *Pandinus imperator,* *Chaerilus tricostatus,* *Androctonus mauritanicus,* *Vespa basalis,* *Vespa lewisii,* *Vespa basalis,* *Polybia paulista,* *Eumenes rubronotatus,* *Walterinnesia aegyptia*	[34, 37, 38, 57, 59, 66, 68, 73, 85, 107]
19	*Shigella flexneri*	Ampicillin, amoxicillin-clavulanic acid, chloramphenicol, tetracycline, trimethoprim, and sulfamethoxazole, nalidixic acid and ciprofloxacin	Mastoparan Mastoparan B	*Vespa basalis,* *Vespa* spp.	[37, 38, 108]
20	*Serratia liquefaciens*	Acylureidopenicillins, ticarcillin, cephalosporins, carbapenems, aztreonam, quinolones and antifolates	Melittin	*Apis mellifera*	[75-80, 109]
21	*Serratia marcescens*	Tetracycline, amoxycillin, amoxycillin/clavulanate and loracarbef	Ponericin (G1, G3, G6, L2, W3-desK, W4, W5, W6) Melittin Opistoporin 1 Hadrurin Parabutoporin Cyto-insectotoxin 1a	*Pachycondyla geoldii,* *Apis mellifera,* *Opistophtalmus carinatus,* *Hadrurus aztecus,* *Lachesana tarabaevi*	[34, 55, 56, 63, 75-80, 109]
22	*Streptococcus pnuemoniae*	Beta-lactams, macrolides, lincosamides, fluoroquinolones, tetracyclines, and trimethoprim-sulfamethoxazole (TMP-SMX)	Opistoporin 1 Parabutoporin Mastoparan - VT5	*Opistophtalmus carinatus,* *Vespa tropica*	[55, 84, 110]
23	*Streptococcus pyogenes*	Erythromycin, clarithromycin	Ponericin (G1, G3, G4, G6, L2, W1, W3-desK, W4, W5, W6) Crotalicidin Batroxicidin Myotoxin (II, III)	*Pachycondyla geoldii,* *Crotalus durissus terrificus,* *Bothrops atrox,* *Bothrops asper*	[34, 36, 111]
24	*Streprococcus sanguinis*	Penicillin, amoxicillin, erythtromycin	Ponericin (G1, G3, G4, G6, L2, W1, 3-desK, W4, W5, W6) Melittin	*Pachycondyla geoldii,* *Apis mellifera*	[34, 75-80, 112]
25	*Shigella sonnei*	Ampicillin, amoxicillin-clavulanic acid, chloramphenicol, nalidixic acid, ciprofloxacin	Mastoparan Mastoparan B Myotoxin (II, III)	*Vespa basalis,* *Vespa basalis,* *Bothrops asper*	[37, 38, 108]
26	*Salmonella typhimurium*	Streptomycin, sulfamethoxazole, tetracycline and ampicillin	Mauriporin Crotamin Myotoxin (II, III)	*Androctonus mauritanicus,* *Crotalus durissus,* *Bothrops asper*	[23, 28, 29, 85, 90, 113]
27	*Yersinia* *enterocolitica*	Ampicillin, amoxicillin/clavulanic acid, and cefazolin	Ponericin (G1, G2) Bactridine (1,2)	*Pachycondyla geoldii,* *Tityus discrepans*	[34, 54, 87]
28	*Mycobacterium tuberculosis*	Rifampicin, isoniazid, floroquinolones, kanamycin, amikacin/capreomycin	VpAmp 1.0 VpAmp 2.0	*Vaejovis punctatus,* *Vaejovis punctatus*	[114, 115]

### Combinational antimicrobial therapies

The potential of animal venoms against antimicrobial resistance has been intensively studied and proven highly effective. However, a combinational approach enables the option of a synergistic mode of action providing preferentially the most effective method for combating resistant bacteria. Enhancement in the activity of commercial antibiotics when administered in combination with venom peptides is already evidenced [11]. Given that animal venoms themselves have been found to exhibit antibiotic properties against many antibiotic-resistant microbes, the potential can be utilized to repurpose commercial antibiotics in treatment of resistant pathogenic microorganisms. Combinational studies are being done in the hope of targeting the resistance mechanisms and getting a better response against the microbial pathogens, which is greater than the sum of their individual effects. Combination therapy is gaining attention over monotherapy from researchers across the globe for many of the life-threatening infectious diseases due to its ability to target multiple facets of a microbial infection [116]. Antimicrobial-venom-based combination drugs can emerge as a research priority due to many advantages over synthetic drugs including rapid clinical usage, increased efficiency, need for lower doses, greater stability, and reduction in side effects as compared to those that arise from the use of commercial antibiotics. The mechanism underlying reduction in antibiotic resistance by different sources of animal venoms is still a puzzle that needs to be resolved. However, it is postulated in some studies that the venom extracts create channels through the plasma membranes of the microbes enabling the distortion of their intracellular components [117, 118].

### Synergism: bright side of antimicrobial studies

Many of the antibiotic combinations have been well studied and established for treatment of the resistant infections. It is inferred that in combination, one drug neutralizes the resistance mechanisms of the bacteria, and repurposes the other drug by increasing its efficacy [116]. There are multiple pathways by which microbes have been successful in resisting antibiotic effects. The countable ones include target site modifications, use of MDR pumps and drug inactivation [119]. A complicated condition is seen when the microbe combines several of these approaches for their protection purpose [120]. The well-studied class of antibiotics like penicillin and chloramphenicol target the microbes by acting upon cell wall synthesis and inhibiting protein synthesis, respectively [121]. These antibiotics are currently facing resistance due to the fact that there are some integral proteins present in the outer cell membrane which act as checkpoints for the entry and exit of antibiotics; when these proteins are either lost or modified, permeability to the antibiotics is altered [120]. Many of the commercial antibiotics have become susceptible to these resistance mechanisms. In recent studies it has been reported that specific venom peptides are capable of inducing perturbations in the cell membranes thus allowing the permeability of antibiotics into bacterial cells [122, 123].

### Recent work on combination therapy

 There are few works that depict the use of venoms in combination with the commercially used antibiotics showing increase in inhibitory activities. In an examination, two antimicrobial peptides (AMPs), denominated La47 and Css54, separated and filtered from the unrefined venom concentrate of spider (*Lachesana* sp*.*) and scorpion (*Centruroides suffusus*), were assessed in combination with the commercial antibiotics, chloramphenicol, ampicillin, novobiocin, streptomycin and kanamycin. Strikingly, blends of La47 with antibiotic agents such as chloramphenicol, streptomycin and kanamycin, showed the best antimicrobial results. Likewise, the other novel peptide Css54 when assessed with respect to antibiotic agents utilized for tuberculosis treatment, isoniazid, rifampicin, pyrazinamide and ethambutol, showed best results with rifampicin[11]. In another study, two bacterial strains that were already resistant to antibiotics, i.e., *S. aureus* and *P. aeruginosa*, were used. There was an improvement observed against the bacterial growth when macropin was given in combination with commercial antibiotic. Combination therapy tried with macropin and antibiotic exhibited antibacterial potential at a lower dose as compared to the peptide or antibiotics used[12] alone. The combination of macropin with gentamycin, tobramycin, ciprofloxacin, levofloxacin, piperacillin or oxacillin, was found to be very effective against the strains of *S. aureus* by inhibiting their growth. Similarly, an additive effect was produced against *P. aeruginosa* strains treated with combinations of macropin and various antibiotics. The combinations of oxacillin with macropin (for *S. aureus*) and piperacillin with macropin (for *P. aeruginosa*) increased the bacteriostasis rate very rapidly indicating a strong inhibitory potential [13]. Overall, these data show a promising outlook for potential clinical treatments of bacterial infections using AMPs and commercial antibiotics. Furthermore, the research interest in venoms for their antibacterial potential has increased with time but no therapeutic approach has yet been achieved in the clinical trial phase.

### Future

In recent times, modulating factors present in animal venoms have been improved with the help of advanced technologies and elucidated with respect to their potential in prevention or minimizing the toxic effects of microbial pathogens. It is believed that bacteria may develop resistance to an animal venom only if a specific target mechanism is involved, similarly to the monotherapy practices carried out in the case of present-day antibiotics. The probability of development of resistance decreases when a mixed array of mechanisms followed by the animal venoms are involved. Given the absence in the literature of any report on bacteria developing resistance against venoms, further research to explore the underlying mechanisms is required.

Until recently, the use of venom for clinical applications was hindered not only by low yield, but also by its complex composition, stability and toxicity aspect, which is still less explored. Given their emergence as significant and novel antimicrobial agents, animal venoms need to be investigated for improvement of treatment by combining these chemical entities with the conventional antibiotics. Enhanced durability, performance, strength, bioavailability can be obtained by the combinational therapeutic approach at the ground level.

## Conclusion

The present situation of multidrug resistance is imposing a serious medical condition around the globe and is continuously augmenting the challenge faced by this line of research. The issue of commercial antibiotic use further becomes complicated due to the augmentation of both reduced efficacy and adverse side effects. This challenge prompted the researchers to seek venom-based antimicrobials as a solution against MDR as they are now known to play a vital role in the individual’s defense mechanism. Antimicrobial constituents, either alone or blended with commercial antibiotics, may prove to be an appropriate option for repurposing the antibiotics used earlier for treating the pathogens but which are ineffective today. The complex antimicrobial mechanism scheme of venoms reduces the defensive ability of bacteria, fungi and viruses and can prove to be helpful in treatment of diseases. The review explores the research in respect of the lethal potential of the venom and the other mechanisms by which animal venoms are able to combat resistance mechanisms. Our goal here was to propose an approach enabling assessment of the impact of combination therapy using a cocktail of animal venoms with the commercial antibiotics. In the literature, many studies have been located using venom for evaluation of their antibacterial potential, but no such studies have been found to date reporting any records of clinical trials. In conclusion, there is an urgent need to promote the venom peptides for clinical trials in order to evaluate their safety and efficacy for combatting resistance mechanisms.
